# Host-Specificity and Dynamics in Bacterial Communities Associated with Bloom-Forming Freshwater Phytoplankton

**DOI:** 10.1371/journal.pone.0085950

**Published:** 2014-01-20

**Authors:** Inessa Lacativa Bagatini, Alexander Eiler, Stefan Bertilsson, Dag Klaveness, Letícia Piton Tessarolli, Armando Augusto Henriques Vieira

**Affiliations:** 1 Laboratório de Ficologia, Departamento de Botânica, Universidade Federal de São Carlos, São Carlos, SP, Brasil; 2 Department of Ecology and Genetics, Limnology and Science for Life Laboratories, Uppsala University, Uppsala, Sweden; 3 Department of Biosciences, University of Oslo, Oslo, Norway; University of Shiga Prefecture, Japan

## Abstract

Many freshwater phytoplankton species have the potential to form transient nuisance blooms that affect water quality and other aquatic biota. Heterotrophic bacteria can influence such blooms via nutrient regeneration but also via antagonism and other biotic interactions. We studied the composition of bacterial communities associated with three bloom-forming freshwater phytoplankton species, the diatom *Aulacoseira granulata* and the cyanobacteria *Microcystis aeruginosa* and *Cylindrospermopsis raciborskii*. Experimental cultures incubated with and without lake bacteria were sampled in three different growth phases and bacterial community composition was assessed by 454-Pyrosequencing of 16S rRNA gene amplicons. *Betaproteobacteria* were dominant in all cultures inoculated with lake bacteria, but decreased during the experiment. In contrast, *Alphaproteobacteria,* which made up the second most abundant class of bacteria, increased overall during the course of the experiment. Other bacterial classes responded in contrasting ways to the experimental incubations causing significantly different bacterial communities to develop in response to host phytoplankton species, growth phase and between attached and free-living fractions. Differences in bacterial community composition between cyanobacteria and diatom cultures were greater than between the two cyanobacteria. Despite the significance, major differences between phytoplankton cultures were in the proportion of the OTUs rather than in the absence or presence of specific taxa. Different phytoplankton species favoring different bacterial communities may have important consequences for the fate of organic matter in systems where these bloom forming species occur. The dynamics and development of transient blooms may also be affected as bacterial communities seem to influence phytoplankton species growth in contrasting ways.

## Introduction

The diatom *Aulacoseira granulata* and the toxic cyanobacteria *Microcystis aeruginosa* and *Cylindrospermopsis raciborskii* are common phytoplankton species in eutrophic freshwater lakes and reservoirs around the world [1]–[3]. These phytoplankton can form transient blooms that greatly impact the energy and nutrient flux in aquatic environments because of the locally high amount of organic matter being produced. This may cause additional problems such as oxygen depletion, and some species may also produce toxins and surface scums [1]. The occurrence and dynamics of phytoplankton blooms can be explained by a combination of abiotic and/or biotic factors [4], [5], where phytoplankton-bacterial interactions may also play a central yet largely overlooked role [6]–[8].

Cellular interactions between microorganisms are important for the dynamics of microbial populations while also influencing microbially mediated biogeochemical cycles [5], [9]. Despite the suggested importance of interactions between freshwater phytoplankton and heterotrophic bacteria, our understanding of such interactions is still very limited [10].

Phytoplankton can interact with free-living bacteria but also maintain attached bacteria on their cellular surface. Such communities may differ significantly in composition from their free-living counterparts [10], [11] and may influence the phytoplankton in contrasting ways [8]. For example, Sher et al. [12] found that bacterial strains could enhance the growth of *Prochlorococcus* by diffusible compounds, but also identified antagonistic effects requiring close cellular proximity. Physical attachment of bacteria to the surface of microalgae may therefore suggest a tight functional association [10], [13]. Nevertheless, free-living bacteria can also be associated to specific microalgae, largely because different phytoplankton species release contrasting spectra of dissolved organic compounds to the surrounding medium [14]. These compounds may then select for certain subsets of the bacterial community capable of efficiently using them to fuel growth [15], [16] or capable to resist against their antibiotic effects [17].

Bacterial communities may also vary with growth phase and physiological status of the microalgae, as shown for experimental cultures [10] or natural phytoplankton blooms [16] and this has been linked to changes in the organic matter released during the different stages of phytoplankton growth [18]. Such community shifts may in turn have feedback effects on phytoplankton growth [12], [19] and stimulate changes in the quality and quantity of compounds that will be released by the algae [18]. Therefore, relationships between bacteria and phytoplankton can be defined according to spatial (proximity of cells) and temporal scales as well as the degree of specificity of the interaction [6].

One important gap in knowledge with regards to microalga-bacteria interactions concerns the taxonomic composition and diversity of bacterial communities associated with distinct algal populations and taxa [10]. Previous studies have revealed the identity of some dominant bacterial taxa that seem to be associated with *M. aeruginosa* (e.g. [20]–[22]). Information on bacterial taxa associated with *Cylindrospermopsis sp*. [22], [23] is on the other hand very limited. For the freshwater diatom *A. granulata* there are no studies focusing on the associated microflora. The available studies are all based on a small number of sequences to describe and compare rather diverse bacterial communities from bloom-forming microalgae. In general, most previous work describing the diversity of bacterial communities associated with freshwater cyanobacterial and marine diatom blooms have been based on less than 150 sequences (e.g. [10], [24]), with the notable exception of Eiler and Bertilsson [20], Berg et al [25], and Li et al [26]. Because NGS (Next Generation Sequencing) approaches typically yield a much larger number of sequences, it enables a more robust comparison between bacterial communities associated with phytoplankton species. Furthermore, the attached bacterial communities can be better resolved despite the high background of phytoplankton-derived sequences.

Using high throughput 454 pyrosequencing of 16S rRNA amplicons, we characterized the bacterial communities associated with three important freshwater phytoplankton species, and tested the hypotheses that (1) bacterial community composition (BCC) is characteristics for different phytoplankton host species, (2) bacterial communities will change gradually during the different phytoplankton growth phases and (3) that there are differences between attached and free-living bacteria in phytoplankton-heterotroph co-cultures.

## Materials and Methods

### Phytoplankton Species and Bacterial Inocula

Two cyanobacterial species, *Microcystis aeruginosa* Kützing (BB005) and *Cylindrospermopsis raciborskii* (Woloszynska) Seenayya & Subba Raju (BB048), and one diatom (Bacillariophyceae) *Aulacoseira granulata* var. *granulata* (Ehrenberg) Simonsen (BB001), were isolated from the hypereutrophic Barra Bonita Reservoir in São Paulo State, Brazil, where they form frequent blooms [27], [28]. The isolates were made axenic and maintained in the culture collection of the Botany Department at the Federal University of São Carlos (World Data Center for Microorganisms No. 835). No specific permission was required for sampling the phytoplankton species and the bacteria studied.

*C. raciborskii* was originally axenic, but was contaminated prior to the experiment. Hence, the culture was washed 3 times by normal centrifugation and once by density gradient centrifugation with Percoll (see below) 2 days before the experiment to minimize bacterial contaminants. The algal inocula were checked for axenic conditions before the experiment in WC (Wright’s Cryptophyte) medium [29] supplemented with peptone and glucose (250 mg L^−1^ of each, WC p+g) and in CPS (casein, peptone and starch) broth and agar media.

A bacterial inoculum was obtained from Barra Bonita Reservoir, on 06^th^ of March-2012. The inoculum consisted of a mixture of equal proportion of the water collected at different depths (0.5, 1, 5, 10 and 15 m), using a sterilized sampling system with a bottle connected to a vacuum pump and an 18 m silicone hose. In the laboratory, the collected water was filtered through 1.2 µm glass fiber filter to remove larger phytoplankton, zooplankton and detritus. Since nanoflagellates can even pass 0.8 µm pores [30] the bacterial inoculum was incubated for 2 hours with cycloheximide (100 mg L^−1^) to kill eukaryotic cells. An aliquot of 0.4 L of the bacterial inoculum was washed by first capturing the bacterial cells on 0.22 µm polycarbonate membrane filters and then rinsing them three times with the same volume of sterile WC medium. The cells were resuspended to the same volume in WC medium and used as bacterial inoculum in the experiment. The bacterial density in the inoculum was ∼1.15×10^7^ cells mL^−1^ and was determined according to Porter and Feig [31]. A fraction (ca. 120 mL) of this inoculum was filtered again through 0.22 µm polycarbonate membrane filters and the filtrate was inoculated in the axenic phytoplankton cultures to serve as controls for the effects of viruses and potentially residual cycloheximide. No flagellates were found in the cultures during the experiment and the effects of any possible remaining cycloheximide was not significant in previous test for *M. aeruginosa* (ANCOVA, F = 2.59 p = 0.12) and *A. granulata* (ANCOVA, F = 0.012, p = 0.91) in liquid culture. The bacteria captured on the polycarbonate membrane were resuspended in WC medium, centrifuged (16000*×g*, 25 min) and stored in −20°C for DNA extraction and sequencing of 16S rRNA genes from the bacterial inoculum.

### Experimental Design

Each exponentially growing phytoplankton species was inoculated in 4 L WC medium [29]. For the diatom, the medium was supplemented with silica (twice the original concentration). The WC medium [29] contains ∼20.7 higher P-PO_4_ and 6.4 times higher N-NO_3_ than concentrations typically found in Barra Bonita Reservoir [27]. Higher nutrient concentration was used to extend the phytoplankton growth curve and obtain sufficient biomass production. All the procedures were done under aseptic conditions. Each culture was immediately split in 2 flasks, and either inoculated with 0.1 L (5% v/v) bacterial inoculum (non-axenic) or amended with a similar volume of the filter-sterilized control medium. Subsequently, each culture was homogenized and 150 mL aliquots were aseptically transferred to 250 mL culture flasks that were incubated at 23±2°C under illumination of 90±10 µmol photons m^−2^ s^−1^ in 12 h:12 h light-dark cycle. For each sampling day, 3 independent replicate flasks each from the bacterial treatment and the controls were analyzed. Sampling dates varied between the phytoplankton species: *Aulacoseira granulata* (days 2, 9 and 16 – early exponential, exponential and stationary phases), *Microcystis aeruginosa* (days 2, 15 and 20 - early exponential, late exponential and stationary phases) and *Cylindrospermopsis raciborskii* (days 2, 15 and 17 - early exponential, stationary and early senescent phases). Controls were checked for axenic condition in WC p+g medium on the same days, except for *C. raciborskii* where only the inoculum was checked and found to be not axenic. These contaminant bacteria in the control cultures were identified. Analyses of chlorophyll-*a* and absorbance were also carried out on the initial day, and absorbance was measured on at least 4 more occasions during the experiment.

### Phytoplankton Growth Analyses

Phytoplankton pigments were extracted using hot 90% ethanol [32]. Chlorophyll-*a* was measured spectrophotometrically and quantified according to Lorenzen [33]. Phytoplankton growth was also followed by *in vivo* chlorophyll absorbance calibrated to extracted chlorophyll-*a* levels.

*In vivo* chlorophyll absorbance for the diatom cultures was measured at 680 nm with correction for particles at 750 nm. For cyanobacterial cultures, the absorbances were also measured at 680 nm, but corrected for particles at 730 nm. These wavelengths were chosen to correlate better with absolute chlorophyll-*a* concentrations (measured as described above) for *A. granulata* (R^2^ = 0.91) and *M. aeruginosa* (R^2^ = 0.98) in previous study.

### Nutrient Concentrations

Nitrate and phosphate were analyzed using Dionex ICS-1100 (Ion Chromatography System, Thermo Scientific) in 0.22 µm filtered samples.

### Scanning Electron Microscopy

Samples obtained during the second sampling were filtered and centrifuged with Percoll (see below). A drop of a suspension containing phytoplankton cells with attached bacteria was then inoculated in fresh WC medium and monthly transferred to new tubes for 2 months. These cultures were used for scanning electron microscopy (SEM) preparation. Control cultures were also maintained for 2 months. A drop of the sample (10 or 20 µL) fixed in 2% glutaraldehyde was deposited onto coverslips, which were previously coated with poly-l-lysine solution [34], and left overnight in a moist chamber. The excess of water was then carefully dried and the coverslips washed twice in distilled water and dehydrated by 10 minutes treatment with increasing ethanol concentrations (1× 50, 70, 80, 90 and 96% and 4× 100% ethanol). Samples were then critical point dried by using liquid carbon dioxide (BalTec CPD 030). The coverslips were mounted onto SEM stubs with carbon sticky tabs and the samples coated with carbon using a Cressington 308UHR sputter coater, before observation in a Hitachi S4800 scanning electron microscope (Hitachi, Tokyo, Japan) operated at 5.0 kV.

### Fraction Separation: Free-living and Attached Bacteria

For the DNA-based analyses, 30 to 40 mL of each culture sample was first aseptically filtered (under a vacuum <200 mmHg) through an 8 µm pore-size cellulose acetate membrane and washed once with 20 mL of sterile WC medium. The free-living bacteria were defined as the fraction <8 µm. None of the phytoplankton species used in the experiment passed through this pore size, and the use of this larger pore size compared to the filter used for the original preparation of the inoculum allowed for a more robust separation of bacteria attached to the phytoplankton cells and their free-living counterparts. The filtrate (free-living bacteria) was centrifuged for 25 min at 16000×*g*, and the pellet maintained frozen until DNA extraction. To recover the phytoplankton-attached bacteria, the fraction retained by the filtration was washed twice with 30 mL of sterile WC medium and resuspended in 2 mL of the same medium using a sterile Pasteur pipette to recover most of the cells. For a better removal of some possible free-living bacteria, the recovered cells were centrifuged in a density gradient with Percoll (GE-HealthCare) in 15 mL falcon tubes with 10 mL of final volume, for 1 h at 15500×*g*, 10°C in a fixed angle-rotor centrifuge (Centrifuge 5804R, Eppendorf). The Percoll concentration was 30% for the Cyanobacteria and 80% for the diatom in WC medium. The unique band for the diatom and the intermediate band for the *Cyanobacteria*, in which there were more cells, were aspirated with a sterile pipette, centrifuged again for 30 min, 16000*×g* in a 1.5 mL tube filled with WC medium, and the pellet stored at -20°C until the DNA extraction. For the second sampling of adhered bacteria from *C. raciborskii* (C_d02_ab) and free-living bacteria of *A. granulata* (A_d02_fb) there were only two replicates for bacterial community analysis. The controls were extracted and analyzed without replicates and without any fractionation procedure: *M. aeruginosa* day 20, *A. granulata* day 16, and all sampling days (02, 15 and 17) for *C. raciborskii* since it was not axenic.

### DNA Extraction, PCR Amplification

DNA extraction was performed as follows: 100 µL of proteinase K solution (50 ng µL^−1^ of proteinase K in TE at pH 8) and 0.2 g of glass beads (150–212 µm, Sigma-Aldrich) were added to each 1.5 mL tube. The samples were incubated in a water bath for 15 min at 45°C and subsequently vortexed at 2500 rpm for 15 s. CTAB and NaCl were added to each tube at a final concentration of 2% CTAB [35] and 1.35 M NaCl. The samples were incubated for 20 min at 65°C and 15 min at 55°C followed by centrifugation at 12000×*g* for 15 min. The supernatant was transferred to a new tube and extracted with phenol:chloroform:isoamyl alcohol (25∶24:1) and chloroform, followed by DNA precipitation with cold 100% ethanol with sodium acetate (final concentration 1 M) and by a washing step with cold 70% ethanol [36]. The DNA was resuspended to 25 µL in TE-4 buffer and the DNA concentration was determined by absorbance at 260 nm on a NanoDrop 2000C spectrophotometer. Partial bacterial 16S rRNA genes (*Escherichia coli* position 341–805, V3–V5 regions) were amplified using universal bacterial primers 341F (CCTACGGGNGGCWGCAG) and 805R (GACTACHVGGGTATCTAATCC) [37]. Primer 341F carried a 454FLX adaptor B at the 5′ end and primer 805R carried a 7-bp molecular barcode specific for each sample followed by a 454FLX adaptor A at the 5′ end [38]. PCR amplification was performed under the following conditions: initial denaturation at 98°C for 30 s, 25 to 29 cycles at 98°C for 10 s, 53°C for 30 s and 72°C for 30 s, and final extension at 72°C for 7 min. Each sample was amplified in duplicate 30 µL reactions using 10–45 ng of template DNA, 0.02 UµL^−1^ of Phusion DNA Polymerase (Finnzymes, Espoo, Finland), 1× Phusion HF buffer, 0.2 mM dNTPs and 0.25 µM of each primer. Each amplification was checked by electrophoresis on a 1% agarose gel [20]. The replicate PCR reactions were pooled and purified using Agencourt AMPure XP PCR purification kit according to manufacturer instructions (Beckman Coulter Inc., Brea, CA, USA). Purified amplicons were quantified using the Quant-iT PicoGreen dsDNA assay Kit (Invitrogen) and pooled in a known concentration to obtain at least 3,000 sequences for the free-living community and 9,000 sequences for attached community of each sample.

### Pyrosequencing and Sequence Analyses

Amplicons from each sample were sequenced from adaptor A on a 454-FLX system using Titanium chemistry (454 Life Sciences, Brandford, CT) at the SNP/SEQ platform hosted by SciLife Lab, Uppsala, Sweden.

From a total of 1,295,767 reads for the total run, 549,029 reads passed quality control and were assigned to the different samples in the present study based on their barcodes (Table S1). The sequences removed were reads from samples not included in this study and ambiguous sequences, i.e. reads with low quality as inferred from their flowcharts and those that did not carry the exact primer sequence. After implementation of these quality control criteria, the remaining reads were denoised using AmpliconNoise Version 1.24 [39]. AmpliconNoise implements algorithms that remove PCR and 454 pyrosequencing noise as well as the chimera removal tool Perseus. Less than 1.1% of the reads were removed by Perseus using settings (alpha = −15, beta = 0.25) for false positive detection, resulting in an average number of 8765 sequences per sample with a range of 2,324 to 16,308 reads per sample (in total 543,428).

To assign reads into OTUs (operational taxonomic units), UCLUST [40] was applied with a 97% sequence similarity cutoff. A representative sequence from each of the resulting OTUs was classified using the naïve Bayesian classifier (Ribosomal Database Project – RDP classifier,) [41] implemented in MOTHUR [42] in combination with the greengenes database gg_OTU_97 and the taxonomy after Hugenholtz [43]. In addition, the reads were annotated against a local freshwater bacterial sequence database (FW) that included almost 12,000 sequence entries [44]. In the text we use the taxonomic placement assigned by naïve Bayesian classifier, followed by the number of the OTU and FW classification in parentheses.

After removal of contaminations as inferred from sequenced blanks as well as phytoplankton reads (cyanobacterial and diatom chloroplasts), 217,653 reads remained with a minimum sequence length of 350 bp length. The complete 454 run has been deposited in the NCBI Short Read Archive under accession number SRR873436.

### Statistical Analyses

All statistical analyses were conducted using R [45]. Phytoplankton growth curves (absorbance) of axenic and non-axenic cultures were compared by Covariance Analysis (ANCOVA) using time as covariate [10].

Treatment effects on bacterial OTUs were visualized in heatmaps [46] after resampling with the perl script daisychopper.pl [47] to 86 sequences, the lowest number of reads for one of the samples. The bacterial heatmap included the 51 most abundant OTUs in the combined dataset.

The Morisita-Horn distance was ordinated in two dimensions using non-metric multidimensional scaling (NMDS) to visualize the dynamics in community structure (β-diversity) using the function metaMDS in R and the none-resampled OTU matrix. This metric was chosen based on its robustness with samples of differing sample size [48], since the number of reads of associated bacteria was low in some samples as a result of high number of reads from *Cyanobacteria* and/or chloroplast. Statistical significance of the differences in community composition between free-living and particle associated bacteria, sampling day and different phytoplankton species (pairwise comparison or between the 3 phytoplankton species) were investigated in an ANOVA experimental design on the basis of the Morisita-Horn distance measure, using permutation methods [49] with the function Adonis in R.

The same analysis was performed using distinct cutoffs of abundance (total proportion of the reads of each OTU in the dataset considering all bacterial reads of the 3 phytoplankton species) and frequency presence (presence of OTUs in the samples). The p-values were corrected by the method of false discovery rate using the function qvalue in R [50].

The library coverage was calculated by Good’s method, using the equation [(1−n/N)×100], where n is number of singletons (unique OTUs) and N is the total number of sequences in a library [51].

## Results

### Growth of Phytoplankton Species and Sampling Days

**Figure 1:**
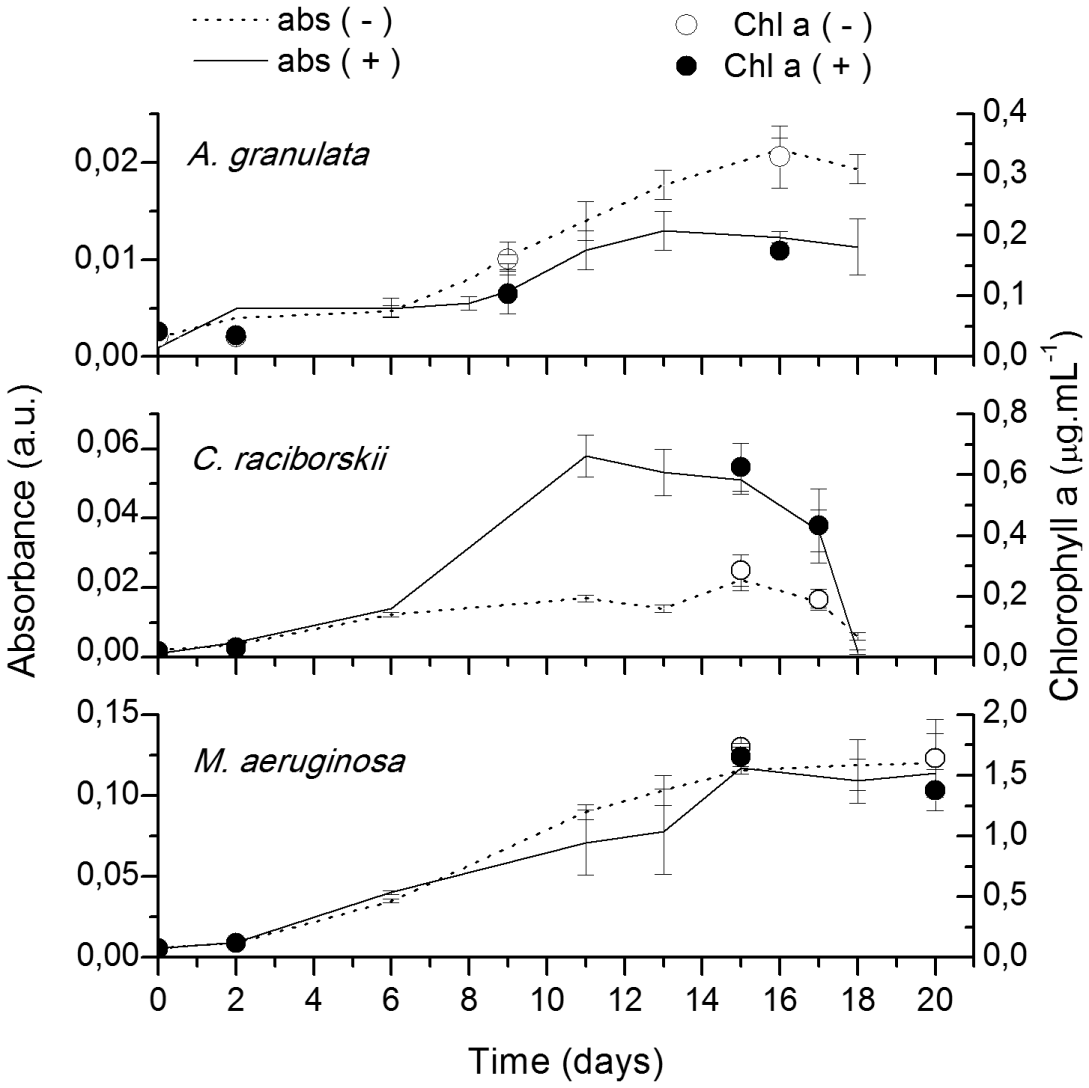
Growth curves of *Aulacoseira granulata, Cylindrospermopsis raciborskii* and *Microcystis aeruginosa*. Lines represent absorbance and symbols represent chlorophyll a concentration. Dashed lines and open symbols are axenic* cultures (−), and solid lines and symbols are non-axenic cultures (+). Error bars represent standard deviation of 3 independent replicates. * *C. raciborskii* was not axenic. Differences in growth curves were significant to *Aulacoseira granulata* (ANCOVA, p<0.001, F = 31.82) and *Cylindrospermopsis raciborskii* (ANCOVA, p<0.001, F = 16.34). No significant effect was observed to *Microcystis aeruginosa* (ANCOVA, p = 0.11, F = 2.62).

There were no significant differences in *M. aeruginosa* growth in response to the bacterial inocula (ANCOVA, p = 0.11, F = 2.62) (Figure 1). In contrast, a significant negative difference in the growth of *A. granulata* (ANCOVA, p<0.001, F = 31.82) was observed (Figure 1). *C. raciborskii* cultures inoculated with lake bacteria exhibited significantly higher growth yield compared to the control (ANCOVA, p<0.001, F = 16.34) (Figure 1). Differently from *M. aeruginosa*, that presented the highest chlorophyll *a* concentrations, both *A. granulata* and *C. raciborskii* did not seem to be nitrate and/or phosphate limited during growth (Table S2).

**Figure 2:**
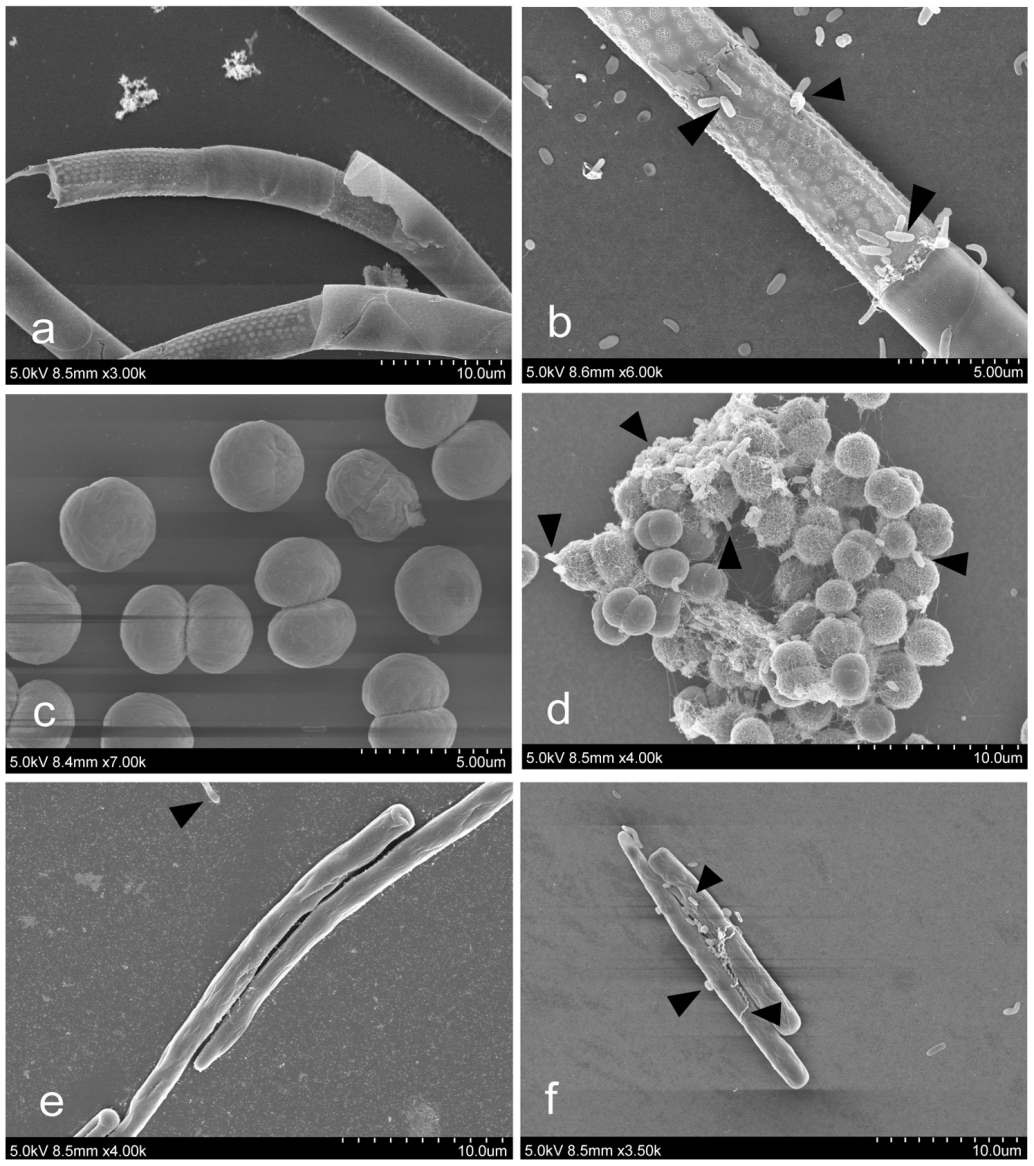
Scanning electron microscopy photomicrograph of the microalgae without (left) and with (right) bacterial inoculum. *Aulacoseira granulata* (a,b), *Microcystis aeruginosa* (c,d) and *Cylindrospermopsis raciborskii* (e,f) cultures without (a, c, e) and with (b,d,f) environmental bacterial inoculum. Black arrows indicate some bacterial (non-phytoplankton) cells.

All three phytoplankton species had bacteria directly attached to the cells. In the case of *M. aeruginosa,* colonies only formed in co-cultures with bacteria, as under this conditions a polysaccharide matrix was produced (Figure 2). *M. aeruginosa* single cells without this polysaccharide layer did not have attached bacteria. Bacteria were not detected by SEM in *M. aeruginosa* and *A. granulata* control cultures (Figure 2a, c). Nevertheless a few reads of non-cyano/chloroplast origin were detected in the controls of *A. granulata* (23 non-chloroplast reads from 10884 reads in total) and *M. aeruginosa* (3 non-cyano reads from 6947 reads in total) (Table S3) that most likely represent contamination during DNA extraction, PCR reactions, library preparation and/or sequencing, but we cannot rule out that they originate from the cultures themselves.

### Reproducibility and Specificity of OTU-phytoplankton Association

Samples were pyrosequenced in triplicate, except for controls (see methods) and the bacterial inoculum. On average 3,753 bacterial 16S rRNA sequences were obtained for each of the 58 samples (range 86–10036 sequences, see Figure S1).

Using a 97% similarity cutoff, a total of 1609 OTUs were obtained for *A. granulata* (552 adhered and 1279 free-living), 1366 for *M. aeruginos*a (134 adhered and 1319 free-living), and 1419 for *C. raciborskii* cultures (279 adhered and 1280 free-living). For the bacterial inoculum we obtained 668 OTUs, implying the presence of other rare OTUs that were not detected in our analysis of the bacterial source community. The library coverage ranged from 94.29% to 99.96% (Figure S2).

Considering only OTUs that occurred in all 3 replicates for each treatment, we obtained 186 OTUs for *A. granulata* with 51 being phytoplankton-specific (see definition of specificity below); 140 for *C. raciborskii,* 25 being specific; and 131 for *M. aeruginosa*, 34 being specific. For the 100 most abundant OTUs for each phytoplankton species (corresponding to more than 92% of the reads of each sample), 79% were detected in each of the triplicates for *C. raciborskii*. The corresponding fraction was 81% for *A. granulata* and 84% for *M. aeruginosa.*

**Figure 3:**
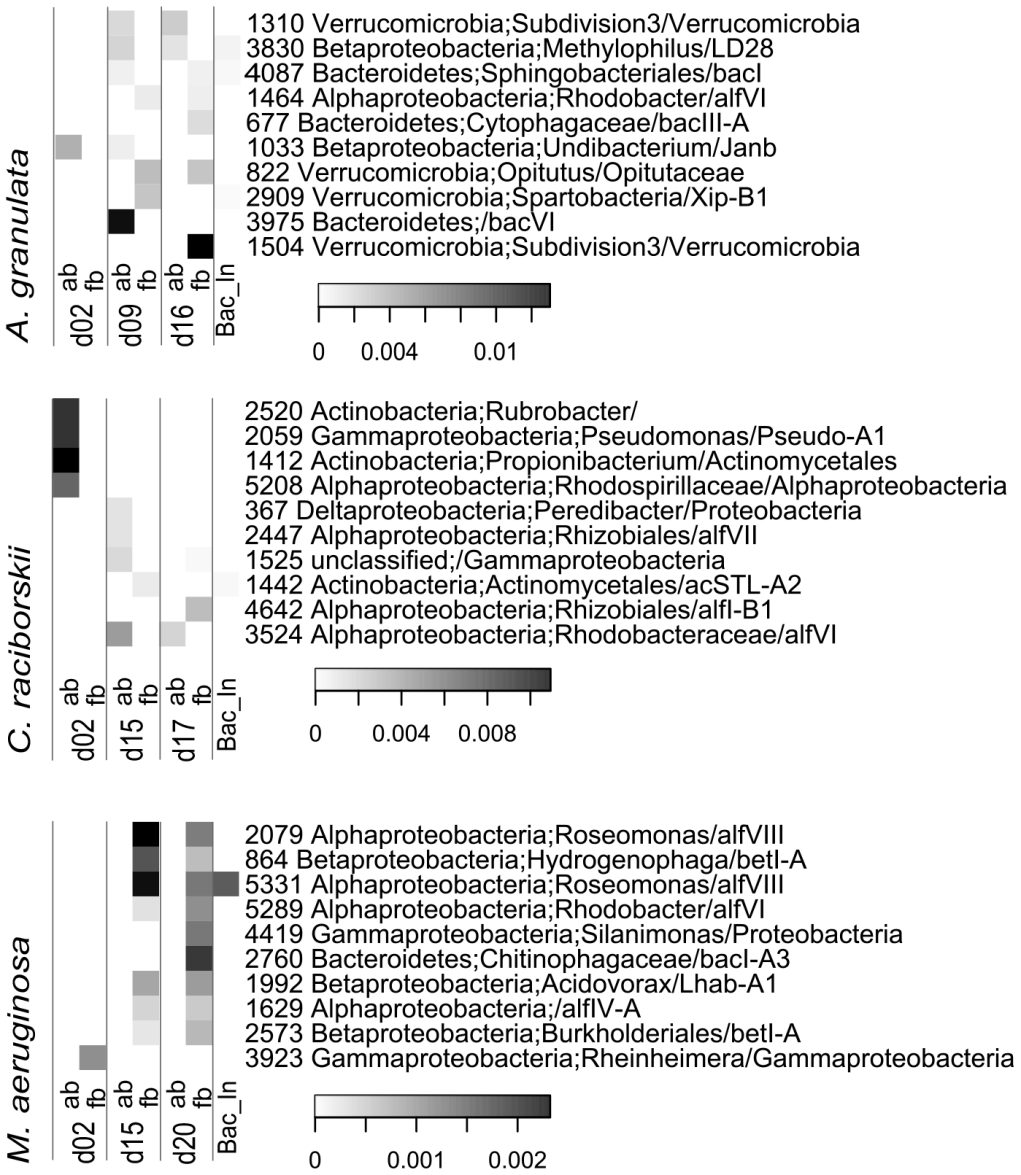
Heatmap of the 10 most abundant specific OTUs associated with each studied phytoplankton species. The non-resampled data was used and only the OTUs that occurred in the 3 independent replicates of one phytoplankton species and that had less than 2 reads in the treatments of the other two were considered specific. Bac_In is the bacterial inoculum; ab indicates attached bacteria and fb, free-living bacteria; dxx indicates the day of sampling. Frequencies are given by relativizing OTUs against the total number of reads of the sample, showing their low proportion. Taxonomic affiliation of two classification databases is shown after identification number: rdp/FW.

Only the OTUs that occurred in the 3 independent replicates of one phytoplankton species and that had a maximum of 2 reads in the treatments of each of the other two were considered specific. Phytoplankton-specific OTUs (Table S4) were typically found in low abundance, each representing less than 1.5% of the total bacterial reads obtained for the sample, and were found in different fractions and growth phases of the three phytoplankton species (Figure 3). All the specific OTUs for *M. aeruginosa* were found in free-living fraction.

### Differences in Bacterial Community: Succession and Selectivity by Host Species and Fraction

**Figure 4:**
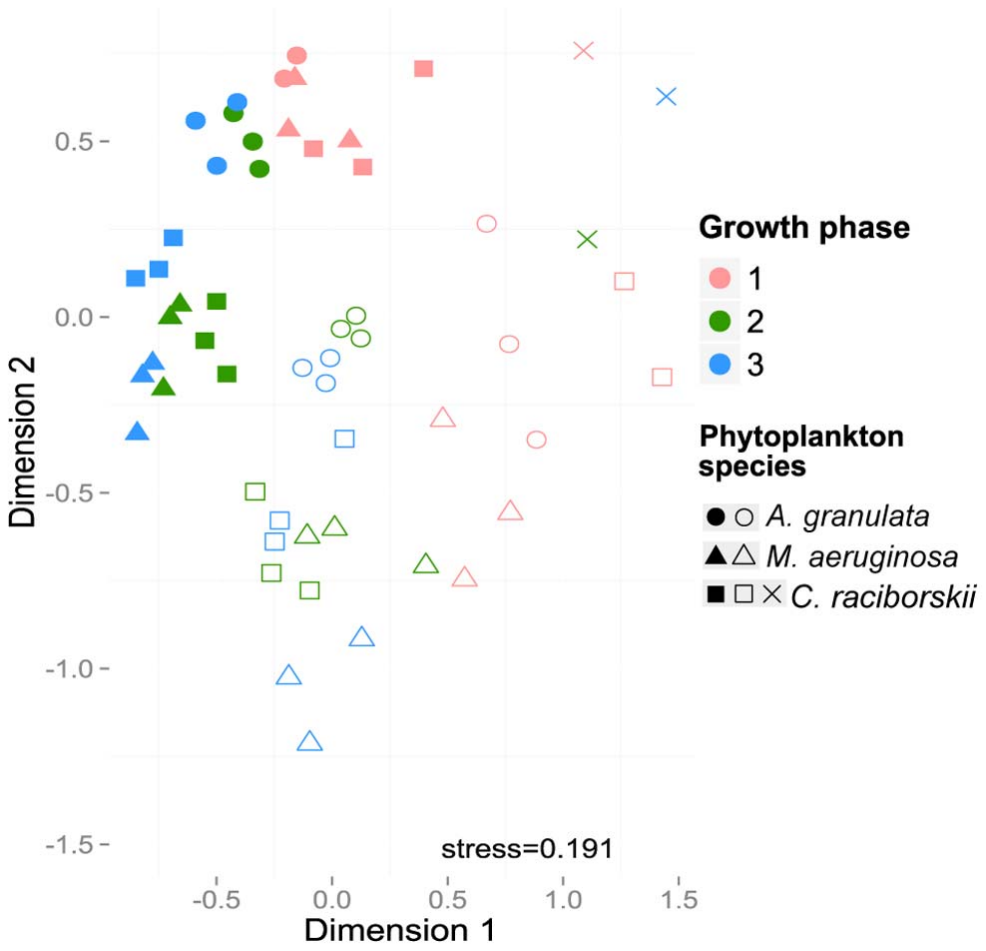
Non-metric multidimensional scaling (NMDS) plot showing differences among bacterial communities by phytoplankton species, fraction and growth phase. Solid and open symbols represent, respectively, free-living and attached communities. The crosses represent *Cylindrospermopsis raciborskii* controls. 1 = lag or beginning of exponential growth phase, 2 * = exponential growth phase, 3* = stationary growth phase. * To *C. raciborskii* 2 and 3 were stationary and senescent phases, respectively.

Bacterial communities differed between the 3 phytoplankton species (Figure 4) for the total bacterial community as well as for the free-living or attached bacteria fractions, and this was supported by PERMANOVA (p-value<0.001) (Table 1). For each species there were significant differences between free-living and particle-attached communities (*M. aeruginosa* p<0.001, *A. granulata* p = 0.002 and, *C. raciborskii* p = 0.003). Differences were also significant between growth phases (Table 1), suggesting a gradual shift in bacterial community composition over time, which is visualized in the NMDS plot (Figure 4).

**Table 1:** Results of Permutational MANOVA comparing bacterial community composition between fraction (attached and free-living), growth phase (age), and phytoplankton species.

		Df	F test	R^2^	p
*Aulacoseira granulata* (A)	fraction	1	15.04	0.31	0.002
	age	2	5.57	0.23	0.005
	fraction*age	2	5.32	0.22	0.002
*Microcystis aeruginosa* (M)	fraction	1	31.12	0.26	<0.001
	age	2	28.50	0.48	<0.001
	fraction*age	2	8.84	0.15	0.002
*Cylindrospermopsis* *raciborskii* (C)	fraction	1	13.70	0.13	0.003
	age	2	38.53	0.70	<0.001
	fraction*age	2	3.81	0.07	0.002
A×M×C	species	2	8.03	0.25	<0.001
A×M×C only attached	species	2	5.57	0.33	0.002
A×M×C only free-living	species	2	6.88	0.37	<0.001
A×M	species	1	9.15	0.22	<0.001
A×C	species	1	10.67	0.25	<0.001
M×C	species	1	4.56	0.12	0.006

Removing less abundant OTUs, by using distinct cutoffs of abundance and occurrence, we obtained higher R^2^ values (Table S5). Still, significant differences were maintained at all cutoffs. PERMANOVA analysis using only the 6 most abundant OTUs (i.e. 3% cutoff), for example, provided an R^2^ of 0.33 (p-value<0.001). The significant differences using only most abundant OTUs reflect the greater differences in the proportion of some taxa, for example, the OTU #4985 (*Sphingobacteria*) made up more than 10% of the reads in *M. aeruginosa* samples and less than 0.5% in the other phytoplankton species samples, whereas OTU #4954 (*Dechloromonas-*related) represented more than 30% of the reads in *A. granulata* and less than 5% in the cyanobacteria.

PERMANOVA analysis also indicated that bacterial communities from cyanobacterial cultures (*M. aeruginosa* × *C. raciborskii*) were more similar among themselves compared to diatom cultures (*A. granulata*), since the R^2^ value was lower among the pairwise comparison of cyanobacterial treatments (Table 1).

**Figure 5:**
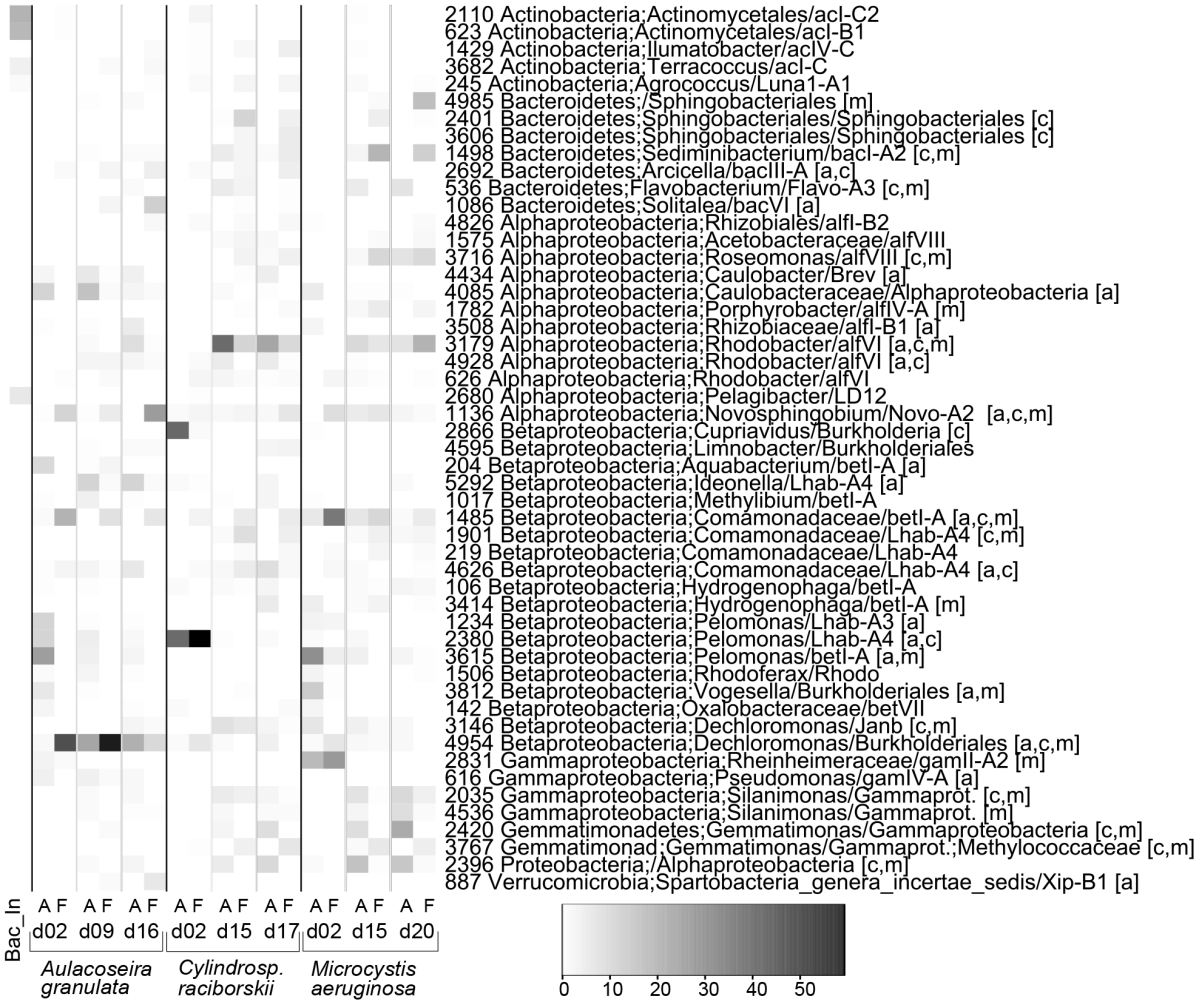
Heatmap displaying the 51 most abundant OTUs after resampling. Taxonomic affiliation of two classification databases is shown after identification number: rdp/FW. *A* and *F* indicate attached and free-living communities, respectively, and dxx indicates day of sampling. Letters in brackets indicate the phytoplankton species in which that OTU was within the most abundant: a, *Aulacoseira granulata* (18 OTUs); c, *Cylindrospermopsis raciborskii* (20 OTUS)*;* m, *Microcystis aeruginosa* (20 OTUs). All these OTUs, except #616, were present in triplicates in at least one treatment of the phytoplankton species where they occurred.

**Figure 6:**
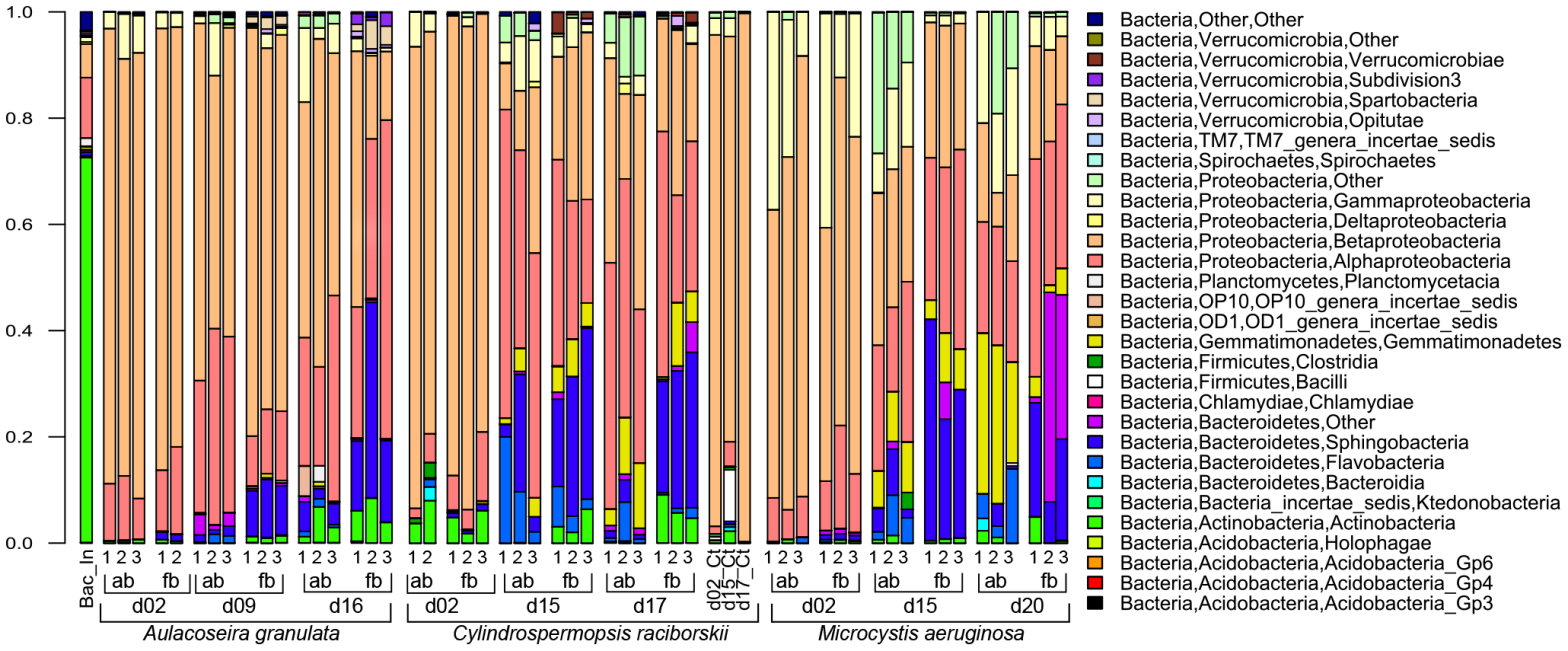
Proportion of bacterial Classes in the replicates of each treatment of the 3 phytoplankton species. Naïve Bayesian classification was used. Bac_In is the bacterial inoculum. Numbers 1–3 represent the replicate, ab and fb indicate attached and free-living communities respectively, Ct is control and dxx is the sampling day.

To visualize the effects of the treatments on OTUs composition while minimizing the influence of sampling depth (Figure 5), the data was resampled using daisychopper.pl. After this, 437 OTUs remained (366 OTUs considering only the treatments). The 51 most abundant OTUs shown in Figure 5 represented from 71 to 99% of the reads of each library in the non-resampled dataset. To display differences in proportion of the main Classes (Figure 6), the non-resampled matrix was used.

Overall, *Betaproteobacteria* was the dominant Class in all incubations (Table 2), but its relative contribution decreased significantly from the first to the last sampling in all treatments (p<0.05) (Table S6). Despite their dominance in all cultures, there were differences in the main OTUs in different phytoplankton species (Figure 5). In contrast, the relative contribution *of Alphaproteobacteria* as the second most abundant class, increased over time, but the significance was variable (Table S6).

**Table 2:** Relative abundance (%) of the main bacterial Classes in the three phytoplankton cultures in different sampling days (d).

	*A. granulata*			*C. raciborskii*			*M. aeruginosa*		
	d02	d09	d16	d02	d15	d17	d02	d15	d 20
Alphaproteobacteria	12±3	22±4	33±15	6±4	38±0	36±11	10±3	28±6	27±4
Betaproteobacteria	82±3	65±7	38±15	84±7	22±9	28±10	63±12	26±2	15±5
Gammaproteobacteria	5±2	2±3	4±4	2±1	5±3	2±01	24±14	7±4	12±2
Flavobacteria	0±0	1±0	1±0	0±0	7±6	2±2	0±0	2±2	3±3
Sphingobacteria	1±0	5±1	13±7	1±0	17±0	14±3	0±0	18±13	9±5

Presented values are mean ± the pooled standard deviation of attached and free-living communities.

The dominance of the Phylum *Proteobacteria* contrasted with the dominant Phylum in the inoculum, in which 70% of the reads were assigned to *Actinobacteria* (Figure 6). Among the 20 most abundant OTUs in the inoculum, one belonged to *Planctomycetes,* 14 belonged to *Actinobacteria* and 5 belonged to *Proteobacteria.* None of these 5 *Proteobacteria* were among the 20 most abundant OTUs in the phytoplankton cultures. Two actinobacterial OTUs (#245 and #1429) were among the most abundant only in *C. raciborskii* cultures.

A major difference at the class level was the higher proportion of *Gemmatimonadetes* in cultures with either of the cyanobacterial species, whereas it was only detected early on the free-living portion of diatom cultures at relative abundances below 0.6% (Figure 6, Table S7).

The proportion of the *Actinobacteria* and *Verrucomicrobia* increased over time in *C. raciborskii* and *A. granulata* cultures, although still representing a small part of the total bacterial community. However, the most abundant OTUs affiliated with these classes were different between the diatom and the cyanobacterial cultures. The *Actinobacteria Agrococcus* (#245, Luna1-A1) and *Ilumatobacter-*related OTUs (#1429, acIV-C) were dominant in *C. raciborskii* cultures, whereas a *Terracoccus-*related (#3682, acIC) was more abundant on diatom cultures (Figure 5).

*Bacteroidetes* was represented mainly by *Sphingobacteria*, which occurred in all cultures (Figure 6). However, the major OTUs differed between diatom and cyanobacteria. The genera *Arcicella* (#2692, bacIII-A) and *Solitalea* (#1086, bacVI) were more abundant in the diatom culture, whereas the genus *Sediminibacterium* (#1498, bacI-A2) and two not identified *Sphingobacteriales* (#2401, #4985) were more abundant in the cyanobacterial cultures (Figure 5). The *Flavobacteria* (also *Bacteroidetes*) presented higher relative abundance in the attached fraction of cyanobacterial cultures.

Another difference between phytoplankton species was the higher proportion of *Gammaproteobacteria* in *M. aeruginosa* cultures. A *Reinheimeraceae* (#2831, gamII-A2) was initially dominant, but was substituted by other *Gammaproteobacteria* as *Silanimonas lenta* (#2035), which was found mainly in the attached fraction of *M. aeruginosa* cultures (Figure 5).

Among the other dominant taxa of other Classes occurring attached to *M. aeruginosa* were the OTUs #2420 (*Gemmatimonas*-related), #2396 (*Alphaproteobacteria*) and #3146 (*Dechloromonas*-related), whereas #1498 (*Sediminibacterium*, bacI-A2), #3179 (*Rhodobacter*-related, alfVI) and #3716 (*Roseomonas,* alfVIII) were most abundant in the free-living fraction.

In *C. raciborskii* the two most abundant OTUs in both fractions were #2380 and #3179. The OTU #2380, a *Pelomonas*, was the main contaminant in the control cultures of *C. raciborskii* (corresponding to more than 84% of total reads for the 3 controls), and its dominance was likely due to its abundance at the 2^nd^ cultivation day, but it was overwhelmed by other OTUs over time, mainly by *Rhodobacter* (alfVI, #3179), predominant in the attached fraction and by some *Sphingobacteriales* (#2401) and *Comamonadaceae* (#1901, Lhab-A4) in the free-living fraction (Figure 5).

The two most abundant OTUs related to the genus *Gemmatimonas* in both cyanobacterial cultures occurred in different fractions: one more abundant in the cyanobacterial-attached community (#2420) and another one in the free-living community (#3767) (Figure 5).

In *A. granulata* cultures, the genera *Arcicella* (#2692, bacIII-A) and *Solitalea* (#1086, bacVI) were more abundant in free-living portion, whereas the *Caulobacteraceae* (#4085, #4434) and the genus *Ideonella* (#5292, Lhab-A4) were more abundant in the attached fractions. The most abundant OTU for *A. granulata* was a *Dechloromonas*-related (#4954, a *Rhodocyclales*), which predominated in both fractions.

## Discussion

### Phytoplankton-specific Bacterial Community Composition

Previous work suggests that the composition of phytoplankton-associated bacterial communities is phytoplankton-specific [10], [15], [22], [52], although there are contrasting results about the specificity of these associations, since they may vary depending on the studied host species [53]. Here we characterized the specific taxonomic groups of bacteria that emerge during different growth stages of bloom forming freshwater phytoplankton species and did so in much greater detail than was previously possible. This deep characterization showed that the BCCs emerging from the same inoculum were characteristic for each phytoplankton species and their growth phases, and differed mainly in the proportion of the OTUs rather than in the presence or absence of specific taxa.

None of the more abundant bacterial phylotypes in the cultures appeared to feature specific associations to any of the studied phytoplankton species during the culturing time used in the experiments. This observation likely would not be possible using less sensitive techniques commonly used in previous studies, as DGGE [10], [22], since we could have found, for example, that the OTU #4985 was specific to *M. aeruginosa,* once it occurred in less than 0.5% in the other phytoplankton cultures.

Although we only followed a single life cycle of each phytoplankton bloom, the variation in the OTUs abundance was strong enough to drive differences in beta-diversity. Furthermore, our results suggest that the observed associations are robust and not merely due to chance, since more than 78% of the most abundant OTUs were present in each replicate of a specific treatment. This is in agreement with Grossart et al. [10] and Schäfer et al. [15] who both found that the community composition in different algal-bacterial co-cultures were reproducible if the same bacterial inoculum was used.

Some degree of specific association can be expected between bacteria and phytoplankton since there is a considerable variation in the contribution of different organic metabolites to the total biomass of different phytoplankton species [14]. Furthermore, different bacteria have distinct capacities to degrading and metabolizing phytoplankton-derived compounds [16] and to resist to antibiotics produced by the phytoplankton [17]. Still, we cannot rule out additional effects resulting from nutrient competition between the phytoplankton species and bacteria, as phytoplankton species are known to differ in their nutrient requirements and uptake kinetics (e.g. [54]) and final concentrations of N and P differed between the phytoplankton treatments (Table S2). However, BCC associated with the two *Cyanobacteria* were more similar among themselves compared to the diatom, despite the higher similarity in nutrient concentrations of *C. raciborskii* and *A. granulata* cultures (Table S2). This may reflect that closely related phytoplankton taxa produce more similar organic compounds [55] subsequently selecting for more similar bacterial communities [22], and may suggest that these compounds were more important on selecting the heterotrophic community than nitrate and phosphate availability.

The expected variation in composition and proportion of organic matter released from viable cells (mainly polysaccharides [3], [14]), is quite likely also reflected in the associated heterotrophic bacterial community. Therefore, as many taxa are shared across our experimental incubations, we speculate that some excreted compounds may overlap between the phytoplankton species and merely vary in abundance. Additionally, polymeric phytoplankton-derived organic matter will not only be processed by the actively hydrolyzing bacteria, but also a wider range of opportunistic bacteria capable of quenching the hydrolysis products will profit [16]. Thus, explaining the observation that differences in BCC between the treatments in the culturing time used in our experiment will be in the proportion rather than in occurrence of OTUs.

More detailed analyses of the bacterial communities revealed a few less abundant phytoplankton-specific OTUs in all treatments (Figure 3 and table S4), with exception for the attached *M. aeruginosa* community. It can be further speculated that these few and low abundant specific OTUs may represent the heterotrophic taxa thriving on species-specific phytoplankton metabolites. However, even using a rigorous definition of specificity, we cannot exclude that sequencing depth influenced the classification into “specific OUT” as non-resampled data were used.

Moreover, we showed that despite major differences in proportion of taxonomic groups, the emerging bacterial community had a different effect on cyanobacterial and diatom growth (Figure 1), suggesting that local bacterial community may play an important role on the dominance of the different phytoplankton species. Although the emerging bacterial community from Barra Bonita reservoir enhanced the growth of *C. raciborskii*, it is not possible to say whether the lake inoculated bacteria directly stimulated the cyanobacterial growth or whether this effect was because they outcompeted the contaminants (mainly genus *Pelomonas/*Lhab-A4) which may have had a negative effect on phytoplankton growth in the control cultures.

### Differences of Groups and OTUs by Phytoplankton Species

Both differences in OTUs proportions and the analysis of higher taxonomic levels revealed differences in associated bacterial communities among phytoplankton species.

Similar proportions (except for *Actinobacteria*) of the dominant groups found associated with *M. aeruginosa* were also found by Eiler and Bertilsson [20] in a naturally occurring *Microcystis*-dominated bloom in eutrophic lake Ekoln. *Proteobacteria* (*Alpha*, *Beta* and to a lesser extent *Gammaproteobacteria*) dominated the associated bacterial communities which also featured an abundance of *Bacteroidetes* and *Actinobacteria.*

To the best of our knowledge, little data about the bacterial groups directly associated with the cosmopolitan and toxic *C. raciborskii*, which has been increasingly reported in cyanobacterial harmful algal blooms [56], are available in the literature. Pope and Patel [24] reported the occurrence of the main Classes found in this work during a bloom dominated by *Aphanizomenon* and *Cylindrospermopsis*, with the exception of *Gemmatimonadetes,* but they sequenced only 44 bacterial 16S rRNA clones. Shi et al [23] reported four bacterial strains associated with *C. raciborskii* maintained in culture, 3 *Burkholderiaceae* (*Betaproteobacteria*) and one *Sphingobacteriales* (*Bacteroidetes*). No data is available for the bloom forming diatom *A. granulata.*

One of the groups found in abundance in cyanobacterial cultures, the *Gemmatimonadetes,* is a minor component among freshwater lake bacteria [44] and scarce reports are available on its occurrence associated with phytoplankton. Shi et al. [21], [57] also found *Gemmatimonadetes* associated with *Microcystis* colonies, but most encounters of this phylum is from soil microbial communities [58].

Another phylum that varied between phytoplankton species was *Actinobacteria*. There was an increase in *actinobacterial* proportion during the stationary growth phase, mainly in *C. raciborskii* and in *A. granulata* cultures. The most abundant genus of this group was *Ilumatobacter*, from clade acIV-C. The tribe Iluma-C1 (an acIV-C) is known to compete successfully when phosphorus levels are elevated [38], and phosphorous was indeed not limiting in the cultures of either of these two phytoplankton species (Table S2). *Actinobacteria* was already observed in the total fraction of associated bacteria during *Cylindrospermopsis* and *Aphanizomenon* blooms [23].

*Verrucomicrobia,* present in *C. raciborskii* and *A. granulata* cultures, are generally observed in abundance during cyanobacterial blooms (e.g. [20], [23], [35]), with less studies reporting their association with diatoms [53], [59]. Members of the Phylum *Bacteroidetes* seem to vary greatly between diatom and cyanobacteria, possibly indicating ecological specialization and adaptation within this diverse phylum.

### Bacterial Community Changes with Phytoplankton Growth Phases

There were significant differences in bacterial populations in the different growth phases in both attached and free-living bacterial communities for the three phytoplankton species. Changes in bacterial community composition during blooms or over time in cultures have already been reported for diatoms, in attached and free-living communities [10], and for cyanobacteria [60]. These studies conclude that succession likely takes place because of differences in the ecophysiology of the phytoplankton, also influenced by differences in nutrient concentration in the cultures [10]. The differences in nutrient concentrations may have indirect (due to influence on phytoplankton) or direct influence on bacterial communities, but are common during phytoplankton blooms [16] and in bath cultures (e.g. [54]).

In a purely qualitative study, Shi et al. [22] reported no difference in the dominant bacterial 16S rRNA DGGE bands during different growth phases of 12 cyanobacterial cultures (among them *M. aeruginosa* and *C. raciborskii*). Also, Schäfer et al. [15] did not observe any differences in BCC when DGGE profiling of 16S rRNA genes was applied to diatom cultures, but Grossart el al. [10] argue that this apparent stability could be due to missing samples from the initial exponential growth phase. As seen in Figures 4–6, exponential and senescent growth phases are more similar than the initial exponential phase, with higher variability in proportion of groups than occurrence of distinct groups.

The most obvious differences in bacterial classes over time in the cyanobacterial cultures were the increasing proportion of *Gemmatimonadetes* in the attached fraction. The only isolated member of this phylum, *Gemmatimonas aurantiaca,* is a slow growing and phosphate accumulating bacteria [61]. The phosphate accumulation could explain its gradually increasing representations in the later stages of the *M. aeruginosa* cultures where nutrients were depleted, but this is most likely not the case for *C. raciborskii* cultures, where phosphate was still available. Thus some compound(s) released by *Cyanobacteria* could have selected for members of this group.

The proportion of *Bacteroidetes* increased towards the end of the experiment. This is not surprising considering that members of the *Sphingobacteria* and *Flavobacteria* are often found in high abundance during periods following phytoplankton blooms [44], either in the surrounding water or attached to phytoplankton [10].

Among the most abundant OTUs during the first day of sampling were several *Comamonadaceae* (*Betaproteobacteria*). As OTUs of this Family were rare in the inoculum, they likely feature rapid growth in response to the experimental manipulation. The clade betI-A (from this Family) is known for their ability to grow fast and can respond rapidly to changing environmental conditions, while their abundance also seem to be positively associated with low-molecular weight alga-derived substrates (see review [44]).

### Differences between Attached and Free-living Bacterial Communities

In agreement with previous studies [10], [52], differences were also observed between attached and free-living communities. The comparison between the phytoplankton-attached and the free-living bacterial communities may to some extent have been influenced by the high density of Cyanobacteria/Chloroplast fragments in the phytoplankton-associated fraction, which caused a shallower characterization of the attached community, despite the deeper sequencing. However, the observed differences were still significant and we could show variations in the relative abundances of some groups between attached and free-living portions. One plausible explanation for this is that the colony and aggregate formation provide a distinct micro-habitat for particle attached bacteria [62], which might select for different functions and different enzymes compared to free-living communities [63]. Furthermore, different phytoplankton species could have fostered bacterial populations with distinct attachment capability and/or provided distinct binding sites (surface characteristics) for these bacterial taxa [59], [64], [65].

The genus *Flavobacterium* (*Bacteroidetes*) was mainly found attached to the *Cyanobacteria*. The *Flavobacteria* seem to play a particularly important role in degradation of complex biopolymers [66], [67] and are also known to feature a copiotroph lifestyle [68], explaining their higher proportion in the attached fraction. Studying marine diatoms, Grossart et al. [10] found that *Flavobacteria* and *Sphingobacteria* dominated the attached community, but in our study, *Sphingobacteria* were instead found in even higher proportions in the free-living bacterial community.

The *Microcystis*-attached community carried a particularly high proportion of *Gemmatimonadetes*. The dominant *Gemmatimonas*-related OTU in this attached community was different from the most abundant OTU in the free-living fraction of both *Cyanobacteria*. In soil, clones affiliated with *Gemmatimonadetes* were overwhelmingly obtained from inner-aggregate fractions [69] and in the biofilm core in stream biofilms [70]. The dominance of different *Gemmatimonas-*related OTUs in different fractions may suggest ecological diversification.

In the diatom cultures, a *Caulobacteraceae* (#4085) was mainly found in the attached community. This family is often found attached to surfaces [71], and *Caulobacter* has previously been reported to occur in close association with *Cryptomonas* [72].

Our results revealed the dynamic development of changing bacterial communities along phytoplankton growth phases and also differences between attached and free-living communities. Using a single bacterial inoculum, we showed that a characteristic bacterial community emerges in response to each type of phytoplankton and present variable effects on their growth. Some of these differences even appear at the class level. Different phytoplankton species and microhabitats clearly favor contrasting subsets of the heterotrophic bacterial community and since different taxa likely represent particular microbial functions [16], this may have important consequences for the carbon flow in aquatic environments after a phytoplankton bloom. The new in depth information on bacterial taxa co-occurring with bloom forming phytoplankton species in attached and free-living portions are however only a first step towards a better understanding of phytoplankton bloom development and dynamics. Future studies should now focus on revealing the mechanisms on how associated bacteria can affect the growth of different phytoplankton species and their bloom formation.

## Supporting Information

Figure S1 **Numbers of non-cyano/chloroplast and of cyano/chloroplast reads, and number of OTUs per sample.** *A. granulata* (A), *C. raciborskii* (C), and *M. aeruginosa* (M) cultures, in different days (dxx) and fractions (ab, adhered bacteria; fb, free-living bacteria).
(TIF)

Figure S2 **Number of singletons and the estimated library coverage (%) per sample.** *A. granulata* (A), *C. raciborskii* (C), and *M. aeruginosa* (M) cultures, in different days (dxx) and fractions (ab, attached bacteria; fb, free-living bacteria).
(TIF)

Table S1 **Barcode sequences used for each sample.** Bac_In, bacterial inoculum. Names of samples are comprised by the first letter of the alga name (A, *Aulacoseira granulata;* C, *Cylindrospermopsis raciborskii,* M, *Microcystis aeruginosa*), day of sampling (dxx), fraction (ab, adhered bacteria; fb, free-living bacteria) and replicate number.
(PDF)

Table S2 **Concentration of dissolved nutrients (mg.L^−1^) in control and treatment cultures (mean±SD).**
(PDF)

Table S3 **Contaminant OTUs in control cultures of** ***Aulacoseira granulata*** **and** ***Microcystis aeruginosa*** **.**
(PDF)

Table S4 **Specific OTUs associated with each studied phytoplankton species.** Taxonomic affiliation of two classification databases is shown after identification number: RDP (Ribosomal database Project) and FW (Freshwater database).
(PDF)

Table S5 **Partial Regression Coefficients (R^2^) from PERMANOVA comparing bacterial community compositions between the three phytoplankton species using different cutoffs.**
(PDF)

Table S6 **Two Sample t-test of** ***Alphaproteobacteria*** **and** ***Betaproteobacteria*** **proportions between sampling days.** Bold values indicate samples where the mean proportion decreased with time. 1 = lag or beginning of exponential growth phase, 2 * = exponential growth phase, 3* = stationary growth phase. * To *Cylindrospermopsis raciborskii* 2 and 3 were stationary and senescent phases, respectively.
(PDF)

Table S7 **Relative abundances of bacterial Classes in each treatment.**
(PDF)
