# Supplementary figures and images for: Host-Specificity and Dynamics in Bacterial Communities Associated with Bloom-Forming Freshwater Phytoplankton
Source: PLoS One. 2014 Jan 20;9(1):e85950. doi: 10.1371/journal.pone.0085950 (PMC3896425; doi:10.1371/journal.pone.0085950)

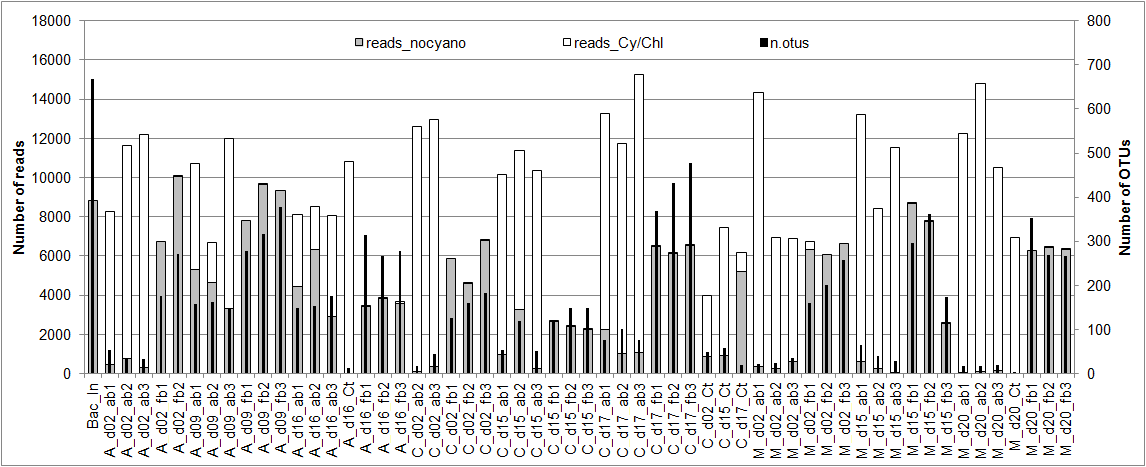

Supplement: Figure S1 — Numbers of non-cyano/chloroplast and of cyano/chloroplast reads, and number of OTUs per sample. A. granulata (A), C. raciborskii (C), and M. aeruginosa (M) cultures, in different days (dxx) and fractions (ab, adhered bacteria; fb, free-living bacteria). (TIF) [file pone.0085950.s001.tif]

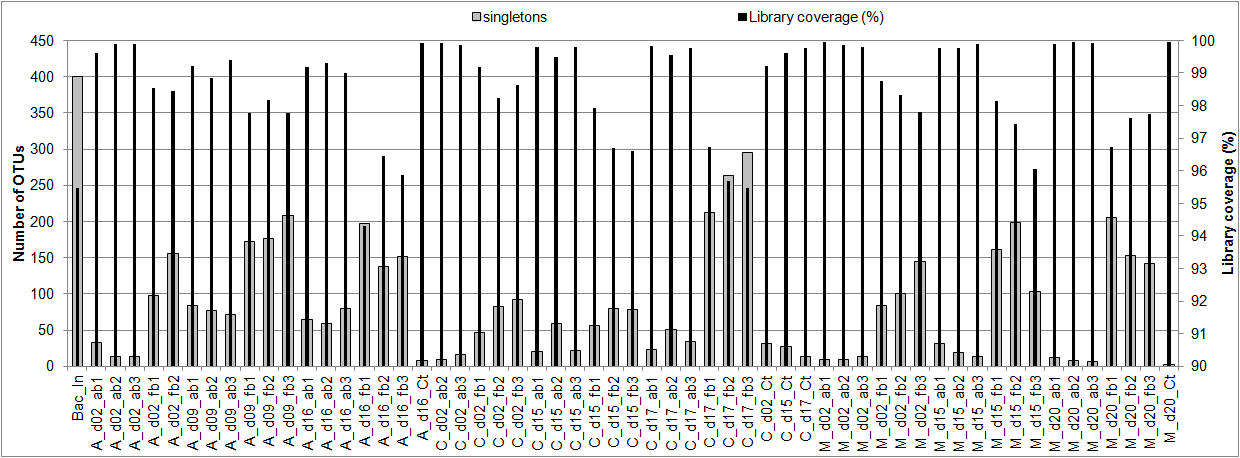

Supplement: Figure S2 — Number of singletons and the estimated library coverage (%) per sample. A. granulata (A), C. raciborskii (C), and M. aeruginosa (M) cultures, in different days (dxx) and fractions (ab, attached bacteria; fb, free-living bacteria). (TIF) [file pone.0085950.s002.tif]
