# Supplementary material for: Host-Specificity and Dynamics in Bacterial Communities Associated with Bloom-Forming Freshwater Phytoplankton
Source: PLoS One. 2014 Jan 20;9(1):e85950. doi: 10.1371/journal.pone.0085950 (PMC3896425; doi:10.1371/journal.pone.0085950)
Supplement: Table S1 — Barcode sequences used for each sample. Bac_In, bacterial inoculum. Names of samples are comprised by the first letter of the alga name (A, Aulacoseira granulata; C, Cylindrospermopsis raciborskii, M, Microcystis aeruginosa), day of sampling (dxx), fraction (ab, adhered bacteria; fb, free-living bacteria) and replicate number. (PDF) [file pone.0085950.s003.pdf]

Table S1. Barcode sequences for each sample

Sample name	Barcode	Sample name	Barcode	Sample name	Barcode
Bac_In	TACTCTC	C_d02_ab2	TAGCACT	M_d02_ab1	TACGCTA
A_d02_ab1	TACTCGA	C_d02_ab3	TAGCAGA	M_d02_ab2	TAGTCAC
A_d02_ab2	TACTGAC	C_d02_fb1	TAGCGTA	M_d02_ab3	TAGACTC
A_d02_ab3	TACTGCA	C_d02_fb2	TCTACTC	M_d02_fb1	TAGACGA
A_d02_fb1	TACGTCA	C_d02_fb3	TCTCTCA	M_d02_fb2	TAGAGAC
A_d02_fb2	TACGAGT	C_d15_ab1	TCACAGT	M_d02_fb3	TAGAGCA
A_d09_ab1	TCTCATC	C_d15_ab2	TCACGTA	M_d15_ab1	TCATCGA
A_d09_ab2	TCTCACT	C_d15_ab3	TCACGAT	M_d15_ab2	TCATGAC
A_d09_ab3	TCTCAGA	C_d15_fb1	TCAGTCA	M_d15_ab3	TCATGCA
A_d09_fb1	TCTGAGT	C_d15_fb2	TCAGATC	M_d15_fb1	TCACTAC
A_d09_fb2	TCATAGC	C_d15_fb3	TCAGAGA	M_d15_fb2	TCACTCT
A_d09_fb3	TCATCTC	C_d17_ab1	TGTCACA	M_d15_fb3	TCACTGA
A_d16_ab1	TCGTAGA	C_d17_ab2	TGTCGTA	M_d20_ab1	TGAGTAC
A_d16_ab2	TCGTGTA	C_d17_ab3	TGTGTCA	M_d20_ab2	TGAGTCT
A_d16_ab3	TCGATCA	C_d17_fb1	TGTGCTA	M_d20_ab3	TGAGTGA
A_d16_Ct	TCAGCTA	C_d17_fb2	TGATCAC	M_d20_fb1	TGAGCAT
A_d16_fb1	TCGACTA	C_d17_fb3	TGACTCA	M_d20_fb2	TGCTAGA
A_d16_fb2	TCGCATA	C_d02_Ct	TAGCTCA	M_d20_fb3	TGCTGTA
A_d16_fb3	TGTACGA	C_d15_Ct	TCACACA	M_d20_Ct	TGACACT
		C_d17_Ct	TGTAGCA		

Barcode sequences used for each sample. Bac_In, bacterial inoculum. Names of samples are comprised by the first letter of the alga name (A, *Aulacoseira granulata;* C, *Cylindrospermopsis raciborskii,* M, *Microcystis aeruginosa*), day of sampling (dxx), fraction (ab, adhered bacteria; fb, free-living bacteria) and replicate number.
