# Supplementary material for: Host-Specificity and Dynamics in Bacterial Communities Associated with Bloom-Forming Freshwater Phytoplankton
Source: PLoS One. 2014 Jan 20;9(1):e85950. doi: 10.1371/journal.pone.0085950 (PMC3896425; doi:10.1371/journal.pone.0085950)
Supplement: Table S2 — Concentration of dissolved nutrients (mg.L−1) in control and treatment cultures (mean±SD). (PDF) [file pone.0085950.s004.pdf]

Table S2. Concentration of dissolved nutrients in control and treatment cultures.

		Control		Treatment	
	**day**	**Nitrate**	**Phosphate**	**Nitrate**	**Phosphate**
A. granulata	2	61.8±0.8	2.9±0.0	64.0±1.2	3.1±0.1
	9	67.5±0.6	3.2±0.0	63.1±5.8	2.9±0.3
	16	64.2±0.5	2.8±0.1	63±1.6	2.6±0.2
M. aeruginosa	2	64.1±0.1	3.2±0.0	66.3±0.2	3.3±0.1
	15	0.1±0.2	0.0	0.1±0.1	0.0
	20	0.1±0.1	0.0	0.0	0.0
C. raciborskii	2	66.0±0.4	3.0±0.1	66.1±0.7	3.0±0.1
	15	55.5±2.3	2.5±0.1	32.5±1.6	1.4±0.1
	17	61.9±1.0	2.7±0.2	40.2±3.6	1.7±0.1

Concentration of dissolved nutrients ($mg.L^{-1}$) in control and treatment cultures of the three host species (mean±SD).
