# Supplementary material for: Host-Specificity and Dynamics in Bacterial Communities Associated with Bloom-Forming Freshwater Phytoplankton
Source: PLoS One. 2014 Jan 20;9(1):e85950. doi: 10.1371/journal.pone.0085950 (PMC3896425; doi:10.1371/journal.pone.0085950)
Supplement: Table S3 — Contaminant OTUs in control cultures of Aulacoseira granulata and Microcystis aeruginosa . (PDF) [file pone.0085950.s005.pdf]

Table S3 – Contaminant OTUs in control cultures of *Aulacoseira granulata* and *Microcystis aeruginosa*.

#OTU	*A. granulata*	*M. aeruginosa*	Classification
1429	2	0	Bacteria;Actinobacteria;Actinobacteria;Ilumatobacter (acIV-C)
3665	1	1	Bacteria;Actinobacteria;Actinobacteria;Actinomycetales;Corynebacterium (acTH2;Myco)
2692	2	-	Bacteria;Bacteroidetes;Sphingobacteria;Sphingobacteriales;Arcicella (bacIII-A)
1415	-	1	Bacteria;Firmicutes;Clostridia;Clostridiales;Veillonellaceae (unclassified)
4051	1	-	Bacteria;Proteobacteria;Alphaproteobacteria;Rhizobiales (alfI)
4044	1	-	Bacteria;Proteobacteria;Alphaproteobacteria;Rhodobacterales;Rhodobacter (alfVI)
3179	1	-	Bacteria;Proteobacteria;Alphaproteobacteria;Rhodobacterales;Rhodobacter (alfVI)
4928	1	-	Bacteria;Proteobacteria;Alphaproteobacteria;Rhodobacterales;Rhodobacter (alfVI)
1136	4	-	Bacteria;Proteobacteria;Alphaproteobacteria;Sphingomonadales;Novosphingobium (Novo)
1506	-	1	Bacteria;Proteobacteria;Betaproteobacteria;Rhodoferax (betI)
4595	1	-	Bacteria;Proteobacteria;Betaproteobacteria;Burkholderiales;Limnobacter (Burkholderiales)
4954	5	-	Bacteria;Proteobacteria;Betaproteobacteria;Rhodocyclales;Dechloromonas (Burkholderiales)
1598	1	-	Bacteria;Proteobacteria;Gammaproteobacteria (LiUU-3-334)
110	1	-	Bacteria;Proteobacteria;Betaproteobacteria;Rhodocyclales;Dechloromonas (Burkholderiales)
854	2	-	Bacteria;Verrucomicrobia;Opitutae;Opitutales;Opitutus (Opitutaceae)
Total (contamination)	23	3	
Chloroplast/ cyanobacterial reads	10861	6944	

Number of reads of contaminant OTUs in control cultures of *Aulacoseira granulata* (16th culturing day) and *Microcystis aeruginosa* (20th culturing day).
