# Supplementary material for: Host-Specificity and Dynamics in Bacterial Communities Associated with Bloom-Forming Freshwater Phytoplankton
Source: PLoS One. 2014 Jan 20;9(1):e85950. doi: 10.1371/journal.pone.0085950 (PMC3896425; doi:10.1371/journal.pone.0085950)
Supplement: Table S4 — Specific OTUs associated with each studied phytoplankton species. Taxonomic affiliation of two classification databases is shown after identification number: RDP (Ribosomal database Project) and FW (Freshwater database). (PDF) [file pone.0085950.s006.pdf]

Table S4 - Specific OTUs associated with each studied phytoplankton species.

Aulacoseira granulata

# OTU	RDP/FW	# OTU	RDP/FW
1033	Betaproteobacteria;Undibacterium/Janb	359	Alphaproteobacteria;/alfI-A1
1132	Betaproteobacteria;Rhodocyclaceae/Burkholderiales	3687	Betaproteobacteria;/betVI
1310	Verrucomicrobia;Subdivision3/Verrucomicrobia	380	Alphaproteobacteria;/M-L-85
1330	Betaproteobacteria;Inhella/Burkholderiales	3826	Betaproteobacteria;Rhodocyclaceae/PnecD
1464	Alphaproteobacteria;Rhodobacter/alfVI	3830	Betaproteobacteria;Methylophilus/LD28
1504	Verrucomicrobia;Subdivision3/Verrucomicrobia	3969	Actinobacteria;Actinomycetales/Actinomycetales
1889	Alphaproteobacteria;Blastomonas/alfIV	3975	Bacteroidetes;/bacVI
1914	Alphaproteobacteria;Rhodobacteraceae/alfVI	3998	Bacteroidetes;Flavobacteriales/bacVI
1988	Betaproteobacteria;Comamonadaceae/Lhab-A4	4053	Betaproteobacteria;Comamonadaceae/betI-A
235	Gammaproteobacteria/Gammaprot.;Methylococcaceae	4078	Alphaproteobacteria;Sphingomonadales/Novo-A2
2371	Bacteroidetes;Sphingobacteriales/bacVI	4087	Bacteroidetes;Sphingobacteriales/bacI
2410	Alphaproteobacteria;Rhodobacter/alfVI	4473	Bacteroidetes;Cytophagaceae/bacIII-A
2422	Betaproteobacteria;Comamonadaceae/Lhab-A4	4548	Alphaproteobacteria;Rhodobacteraceae/alfVI
2495	Betaproteobacteria;Pelomonas/Burkholderiales	4624	Gammaproteobacteria;Rheinheimera/Sphingomonadaceae
2560	Betaproteobacteria;Comamonadaceae/Lhab-A4	4698	Bacteroidetes;/bacVI
2610	Betaproteobacteria;Pseudacidovorax/betI-A	4868	Gammaproteobacteria;/Gammaprot;Methylococcaceae
2666	Alphaproteobacteria;Caulobacter/alfVI	4994	Alphaproteobacteria;Sphingomonadaceae/M-L-85
2881	Bacteroidetes;Chitinophagaceae/bacI-B1	5171	Betaproteobacteria;Ideonella/Lhab-A4
2909	Verrucomicrobia;Spartobacteria/Xip-B1	5199	Gammaproteobacteria;Rheinheimera/Gammaproteobacteria
2976	Verrucomicrobia;Subdivision3/Verrucomicrobia	666	Bacteroidetes;Sphingobacteriales/Sphingobacteriales
3037	Alphaproteobacteria;Blastomonas/alfIV-B	677	Bacteroidetes;Cytophagaceae/bacIII-A
3056	Bacteroidetes;Cytophagaceae/bacIII-A1	694	Alphaproteobacteria;Rhizobiales/alfI-A1
3100	Betaproteobacteria;Vogesella/Burkholderiales	765	Betaproteobacteria;Herbaspirillum/betVII-B1
3228	Betaproteobacteria;Vogesella/Burkholderiales	822	Verrucomicrobia;Opitutus/Opitutaceae
3357	Alphaproteobacteria;/Pyxis	966	Betaproteobacteria;Comamonadaceae/alfIV
3576	Bacteroidetes;Chitinophagaceae/bacI-A2		

Microcystis aeruginosa

# OTU	RDP/FW	# OTU	RDP/FW
1227	Alphaproteobacteria;Acetobacteraceae/alfVIII	4419	Gammaproteobacteria;Silanimonas/Proteobacteria
1550	Bacteroidetes;Cloacibacterium/bacVI	450	Bacteroidetes;Sphingobacteriales/bacI
1629	Alphaproteobacteria;/alfIV-A	4507	Proteobacteria;/Burkholderiales
1673	Alphaproteobacteria;Sphingomonadales/Novo-A2	4585	Alphaproteobacteria;Acetobacteraceae/alfVIII
1992	Betaproteobacteria;Acidovorax/Lhab-A1	4810	Betaproteobacteria;Rhodocyclaceae/Burkholderiales
2079	Alphaproteobacteria;Roseomonas/alfVIII	4998	Betaproteobacteria;Comamonadaceae/betI-A
2123	Proteobacteria;/alfVI	5109	Betaproteobacteria;Comamonadaceae/betI
2573	Betaproteobacteria;Burkholderiales/betI-A	5250	Alphaproteobacteria;/alfVI
2760	Bacteroidetes;Chitinophagaceae/bacI-A3	5289	Alphaproteobacteria;Rhodobacter/alfVI
2886	Betaproteobacteria;Burkholderiales/Betaproteobacteria	5301	Alphaproteobacteria;Erythrobacteraceae/Novo-A1
3013	Betaproteobacteria;Burkholderiales/Lhab-A4	5331	Alphaproteobacteria;Roseomonas/alfVIII
3105	Gammaproteobacteria;Rheinheimera/Proteobacteria	5359	Alphaproteobacteria;Acetobacteraceae/alfVIII
355	Gemmatimonadetes;Gemmatimonas/Proteobacteria	5395	Alphaproteobacteria;Porphyrobacter/alfIV-A
3923	Gammaproteobacteria;Rheinheimera/Gammaproteobacteria	639	Gemmatimonadetes;Gemmatimonas/Gammaproteobacteria
4149	Alphaproteobacteria;Erythromicrobium/Novo-A1	717	Gemmatimonadetes;Gemmatimonas/Gammaproteobacteria
423	Gammaproteobacteria;Silanimonas/Proteobacteria	864	Betaproteobacteria;Hydrogenophaga/betI-A
4393	Alphaproteobacteria;Rhodobacteraceae/alfVI	925	Alphaproteobacteria;Acetobacteraceae/alfVIII

Cylindrospermopsis raciborskii

# OTU	RDP/FW	# OTU	RDP/FW
1182	Bacteroidetes;Algoriphagus/Algor	3368	Betaproteobacteria;Burkholderiales/Lhab-A4
1272	Betaproteobacteria;Oxalobacteraceae/betVII-B1	3484	Alphaproteobacteria;Rhodobacteraceae/M-L-85
1412	Actinobacteria;Propionibacterium/Actinomycetales	3524	Alphaproteobacteria;Rhodobacteraceae/alfVI
1442	Actinobacteria;Actinomycetales/acSTL-A2	3632	Bacteroidetes;Cytophagaceae/bacIII-A
1525	unclassified;/Gammaproteobacteria	367	Deltaproteobacteria;Peredibacter/Proteobacteria
1936	Bacteroidetes;Sphingobacteriales/Algor	3993	Gammaproteobacteria;Alteromonadales/gamII-A2
2059	Gammaproteobacteria;Pseudomonas/Pseudo-A1	4642	Alphaproteobacteria;Rhizobiales/alfI-B1
2168	Alphaproteobacteria;Bosea/alfI-A1	4758	Actinobacteria;Actinomycetales/Luna1-A1
2190	Betaproteobacteria;Aquabacterium/Burkholderiales	4949	Actinobacteria;/Actinobacteria
2447	Alphaproteobacteria;Rhizobiales/alfVII	4965	Bacteroidetes;Sphingobacteriales/Sphingobacteriales
2520	Actinobacteria;Rubrobacter/	5208	Alphaproteobacteria;Rhodospirillaceae/Alphaproteobacteria
2558	Alphaproteobacteria;Rhodobacteraceae/alfVI	687	Bacteroidetes;Flavisolibacter/bacI-A
3165	Bacteroidetes;Cytophagaceae/bacIII-A		

Specific OTUs associated with each studied phytoplankton species. Taxonomic affiliation of two classification databases is shown after identification number: RDP (Ribosomal database Projetct) and FW (Freshwater database).
