# Supplementary material for: Host-Specificity and Dynamics in Bacterial Communities Associated with Bloom-Forming Freshwater Phytoplankton
Source: PLoS One. 2014 Jan 20;9(1):e85950. doi: 10.1371/journal.pone.0085950 (PMC3896425; doi:10.1371/journal.pone.0085950)
Supplement: Table S5 — Partial Regression Coefficients (R2) from PERMANOVA comparing bacterial community compositions between the three phytoplankton species using different cutoffs. (PDF) [file pone.0085950.s007.pdf]

Table S5 – Partial Regression Coefficients (R^2) from PERMANOVA comparing bacterial community compositions between the three phytoplankton species using different cutoffs.

**Cutoff proportion (remained OTUs)**	**R^2**
>0,001% (1099)	0.253
>0,01% (265)	0.254
>0,1% (81)	0.255
>0,5% (33)	0.264
>1% (18)	0.276
>2% (9)	0.299
>3% (6)	0.333
**Cutoff frequency (remained OTUs)**	
>3 samples (554)	0.254
>10 samples (125)	0.256
>15 samples (75)	0.258
>20 samples (51)	0.257
>25 samples (29)	0.271
>40 samples (6)	0.318

Partial Regression Coefficients (R^2) from Permutational MANOVA comparing bacterial community compositions between the three phytoplankton host species using different cutoffs to exclude less abundant (based on proportion of reads of each OTU in relation of total bacterial reads of analyzed samples) or less frequent OTUs (frequency of presence in the samples). Numbers of remained OTUs after the cutoff are between brackets. All the p-values were significant (<0.001).
