# Supplementary material for: Host-Specificity and Dynamics in Bacterial Communities Associated with Bloom-Forming Freshwater Phytoplankton
Source: PLoS One. 2014 Jan 20;9(1):e85950. doi: 10.1371/journal.pone.0085950 (PMC3896425; doi:10.1371/journal.pone.0085950)
Supplement: Table S6 — Two Sample t-test of Alphaproteobacteria and Betaproteobacteria proportions between sampling days. Bold values indicate samples where the mean proportion decreased with time. 1 = lag or beginning of exponential growth phase, 2 * = exponential growth phase, 3* = stationary growth phase. * To Cylindrospermopsis raciborskii 2 and 3 were stationary and senescent phases, respectively. (PDF) [file pone.0085950.s008.pdf]

Table S6 – Two Sample t-test of *Alphaproteobacteria* and *Betaproteobacteria* proportions between sampling days.

		**Alphaproteobacteria**			**Betaproteobacteria**		
		1-2	2-3	1-3	1-2	2-3	1-3
Attached	*A. granulata*	0.017	**Ns**.	Ns.	**0.035**	**Ns.**	**0.018**
	M. aeruginosa	Ns.	**Ns.**	<0.001	**0.037**	**Ns.**	**0.012**
	C. raciborskii	0.013	Ns.	0.016	**0.006**	Ns.	**0.014**
Free-living	*A. granulata*	**Ns.**	Ns.	Ns.	**Ns.**	**0.049**	**0.038**
	M. aeruginosa	0.014	Ns.	0.022	**0.025**	**Ns.**	**0.009**
	C. raciborskii	Ns.	Ns.	Ns.	**<0.001**	**Ns.**	**<0.001**

P-values from Welch Two Sample t-test of *Alphaproteobacteria* and *Betaproteobacteria* proportions between sampling days. Bold values indicate samples where the mean proportion decreased with time. 1 = lag or beginning of exponential growth phase, 2 *=exponential growth phase, 3* = stationary growth phase. * To *Cylindrospermopsis raciborskii* 2 and 3 were stationary and senescent phases, respectively
