# Supplementary material for: Host-Specificity and Dynamics in Bacterial Communities Associated with Bloom-Forming Freshwater Phytoplankton
Source: PLoS One. 2014 Jan 20;9(1):e85950. doi: 10.1371/journal.pone.0085950 (PMC3896425; doi:10.1371/journal.pone.0085950)
Supplement: Table S7 — Relative abundances of bacterial Classes in each treatment. (PDF) [file pone.0085950.s009.pdf]

Table S7 - Relative abundances of Classes in each treatment.

Taxon	Bac_In	A_d02_ab	A_d02_fb	A_d09_ab	A_d09_fb	A_d16_ab	A_d16_fb	C_d02_ab	C_d02_fb	C_d15_ab	C_d15_fb	C_d17_ab	C_d17_fb	C_Ct	M_d02_ab	M_d02_fb	M_d15_ab	M_d15_fb	M_d20_ab	M_d20_fb
Bacteria,Acidobacteria,Acidobacteria_Gp3	-	-	-	-	0.06±0.05	0.09±0.04	0.13±0.17	-	-	-	-	0.03±0.05	0.03±0.01	-	-	-	-	-	-	-
Bacteria,Acidobacteria,Acidobacteria_Gp4	0.11	-	-	-	-	-	-	-	-	-	-	-	-	-	-	-	-	-	-	-
Bacteria,Acidobacteria,Acidobacteria_Gp6	-	-	-	-	0±0.01	-	0.01±0.02	-	-	-	-	-	-	-	-	-	-	0±0.01	-	-
Bacteria,Acidobacteria,Holophagae	0.01	-	-	-	-	-	-	-	-	-	-	-	-	-	-	-	-	-	-	-
Bacteria,Actinobacteria,Actinobacteria	72.4	0.43±0.28	0.48±0.14	0.04±0.04	1.11±0.16	3.58±2.83	6.02±2.31	5.87±3.01	4.25±2.21	0.03±0.05	3 .83±2.27	0.16±0.15	6.47±2.28	0.92±1.18	0.25±0.43	0. 4±0.19	0.7±0.72	0.72±0.27	1.13±1.16	1.79±2.71
Bacteria,Bacteria_incertae_sedis,Ktedonobacteria	0.02	-	-	-	-	-	0.01±0.02	-	-	-	-	0.05±0.05	0.01±0.01	-	-	-	-	-	-	-
Bacteria,Bacteroidetes,Bacteroidia	-	-	-	-	-	-	-	1.29±1.82	-	-	-	-	-	0.28±0.42	-	-	-	-	0.78±1.34	-
Bacteria,Bacteroidetes,Flavobacteria	0.33	-	0.02±0.01	1.03±0.8	0.13±0.05	1±0.51	0.05±0.04	0.71±1.01	0.01±0.02	10.57±8.98	4.1 7 ±3	2.91±3.82	1.05±0.8	0.22±0.28	0.46±0.57	0.22±0. 2	4.6±3.06	0.03±0.01	6.92±6.24	0.06±0.06
Bacteria,Bacteroidetes,Sphingobacteria	0.68	-	1.24±0.13	1.34±0.49	9.57±1.21	3.8±1.86	21.74±13.1	0.14±0.2	0.83±0.33	9.09±11.27	2 4.96±7.94	2.12±1.81	25.37±4.18	0.18±0.31	-	0.96±0.1	4.89±3.56	30.7±9.86	1.6±2.31	16.07±7.33
Bacteria,Bacteroidetes,Other	0.05	-	0.09±0.03	2.42±1.53	0.43±0.1	0.62±0.39	0.38±0.09	-	0.09±0.04	0.22±0.29	0.56±0.67	1.1±0.1	2.34±2.91	-	-	0.75±0.21	0.48±0.83	2.33±3.98	-	22.56±19.58
Bacteria,Chlamydiae,Chlamydiae	0.38	-	0.01±0.01	-	-	-	-	-	0.02±0.02	-	-	-	-	-	-	0.01±0.01	-	-	-	-
Bacteria,Firmicutes,Bacilli	0.07	-	-	0.01±0.01	-	-	-	-	0.01±0.01	-	-	-	-	3.43±5.44	-	-	-	-	0.19±0.32	-
Bacteria,Firmicutes,Clostridia	-	0.13±0.23	-	-	-	-	-	1.9±1.36	0.07±0.07	-	-	-	-	0.41±0.26	-	-	1.11±1.79	-	-	-
Bacteria,Gemmatimonadetes,Gemmatimonadetes	0.49	-	0.02±0.02	0.04±0.02	0.55±0.2	0.3±0.47	0.16±0.11	-	0.26±0.23	3 .01±1.69	5.42±1.39	8.66±4.91	6.03±5.74	-	-	0.02±0.0 1	8.59±1.5	6.82±2.93	26.34±6.36	3.37±1.85
Bacteria,OD1,OD1_genera_incertae_sedis	0.15	-	-	-	-	-	-	-	0.02±0.01	-	-	-	-	-	-	-	-	-	-	-
Bacteria,OP10,OP10_genera_incertae_sedis	-	-	-	0.08±0.14	-	1.9±3.23	0.01±0.02	-	-	-	0.03±0.04	-	0.01±0.01	0.04±0.06	-	-	-	-	-	-
Bacteria,Planctomycetes,Planctomycetacia	1.53	-	-	-	-	1.01±1.72	0.01±0.02	-	0.02±0.02	-	0.01±0.02	-	-	-	-	0.01±0.01	-	0±0.01	-	-
Bacteria,Proteobacteria,Alphaproteobacteria	11.38	10.18±2.27	14.74±2.8	31.65±6.17	11.53±1.9	27.18±10.4	38.22±19.0	3.65±2.52	7.74±4.75	47.16±10. 4	28.12±9.84	40.08±9.68	31.58±13.3	2.01±2.38	7.13±1.41	13.24±5.3 9	23.25±7.15	31.84±5.43	20.76±1.68	32.97±7.21
Bacteria,Proteobacteria,Betaproteobacteria	6.31	82.71±3.73	80.43±2.4	57.68±9.8	71.89±4.5	50.56±9.69	25.58±19.6	81.32±7.92	85.39±6.2	17.02±12.4	26.56±6.37	31.64±13.56	23.53±6.7	89.44±11.91	67.88±14.41	58.9±9.74	26.66±1.7	25.29±1.48	13.73±6.47	17.11±4.21
Bacteria,Proteobacteria,Deltaproteobacteria	0.4	-	-	0.21±0.34	0.46±0.71	0.03±0.03	-	-	0.02±0.02	0.43±0.55	0.04±0.04	0.65±1. 13	0.1±0.1	0.01±0.01	-	-	0.05±0.09	0.01±0.01	-	0.01±0.01
Bacteria,Proteobacteria,Gammaproteobacteria	0.9	6.19±2.74	2.8±0.18	3.84±5.27	1.09±1.37	7.16±6.12	1.18±1.07	4.99±2.2	0.7±0.88	7.27±3.3	3.61±2.01	2.6±1.22	1.33±1.67	2.27±1.79	23.78±14.59	25.1 6±14.28	12.8±4.73	1.71±0.35	18.65±3.27	5.16±1.31
Bacteria,Proteobacteria,Other	0.26	0.16±0.18	0.13±0.05	0.95±0.78	0.06±0.02	2.2±0.25	0.12±0.12	-	0.31±0.44	3.73±1.73	0.46±0.21	9.31±3.26	0.27±0.15	0.75±0.64	0.49±0.85	0.31±0.06	16.81±8.72	0.37±0.19	9.92±9.59	0.76±0.1
Bacteria,Spirochaetes,Spirochaetes	0.01	-	-	-	-	0.01±0.02	0.01±0.02	-	-	-	-	-	-	-	-	-	-	-	-	0.01±0.01
Bacteria,TM7,TM7_genera_incertae_sedis	0.09	-	-	-	0±0.01	0.02±0.03	-	-	0±0.01	-	-	-	-	-	-	-	-	0±0.01	-	-
Bacteria,Verrucomicrobia,Opitutae	0.41	-	-	0.27±0.13	0.51±0.27	0.07±0.01	0.78±0.26	0.14±0.2	0.04±0.03	0.47±0.82	0.32±0.45	-	0.87±0.91	-	-	-	-	0.01±0.01	-	-
Bacteria,Verrucomicrobia,Spartobacteria	0.34	-	-	0.01±0.01	1.72±0.44	0.03±0.04	3.41±2.06	-	0.02±0.03	-	-	-	-	-	-	-	-	-	-	0.01±0.01
Bacteria,Verrucomicrobia,Subdivision3	-	-	-	0.2±0.11	0.05±0.03	0.32±0.23	1.73±1.06	-	-	-	-	-	0.01±0.02	-	-	-	-	-	-	-
Bacteria,Verrucomicrobia,Verrucomicrobiae	0.21	-	-	0.11±0.09	0.25±0.12	-	0.02±0.02	-	0.02±0.03	-	1.87±1.84	0.32±0.25	0.88±0.89	-	-	-	-	0.01±0.01	-	0.04±0.06
Bacteria,Verrucomicrobia,Other	0.01	-	-	-	0.01±0.01	-	0.04±0.08	-	0±0.01	-	-	-	-	-	-	-	-	-	-	-
Bacteria,Other,Other	3.43	0.2±0.18	0.04±0.01	0.13±0.15	0.57±0.17	0.1±0.05	0.39±0.39	-	0.17±0.17	1.01±1.01	0.04±0.04	0.38±0.19	0.13±0.04	0.04±0.07	-	0.03±0.03	0.05±0.09	0.14±0.03	-	0.09±0.07

Relative abundances (% of reads per sample) of Classes in the replicates of each treatment. Naïve bayesian classification was used. Bac_In, bacterial inoculum. Names of samples are comprised by the first letter of the alga name (A, *Aulacoseira granulata;* C, *Cylindrospermopsis raciborskii,* M, *Microcystis aeruginosa*), day of sampling (dxx), fraction (ab, adhered bacteria; fb, free-living bacteria). Mean ±SD, n = 3. – denotes not detected.
